# Designated and non-designated trauma centers and trauma patients: a retrospective analysis of non-fatal trauma discharges in Georgia, 2021

**DOI:** 10.1186/s40621-026-00665-6

**Published:** 2026-02-25

**Authors:** Biplav Babu Tiwari, Eunhae Shin, Nemin Wu, Lan Mu, M. Mahmud Khan, Elizabeth Atkins, Elizabeth Benjamin, Janani Rajbhandari

**Affiliations:** 1 Department of Health Policy and Management, College of Public Health, The University of Georgia, Athens, GA 30602 USA; 2 Department of Geography, Franklin College of Arts and Sciences, The University of Georgia, Athens, GA 30602 USA; 3 Georgia Trauma Commission, Madison, GA 30650 USA; 4 Grady Health System, Atlanta, GA 30603 USA

**Keywords:** Trauma care, Designated trauma centers, Non-trauma centers, Geographical access, proximity to trauma centers, Injury severity

## Abstract

**Background:**

Directing trauma patients to Designated Trauma Centers (DTCs) for treatment of trauma injury is vital to ensure patients’ access to quality trauma care. We study injury severity and distance to DTCs as major factors associated with care and discharge of trauma patients.

**Methods:**

We conducted a retrospective cross-sectional study using 2021 de-identified non-fatal hospital discharge data from Georgia, identifying 225,389 unique adult trauma patients with single discharge records. The outcome was the discharging hospital’s trauma designation. Predictors included whether the nearest hospital was DTC, road network distance to the nearest DTC, and the International Classification of Diseases-based Injury Severity Score (ICISS). Logistic regression with backward stepwise selection identified predictors of DTC vs. Non-designated Trauma Center (NTC) discharge, adjusting for sociodemographic, health, and injury characteristics.

**Results:**

Patients discharged from DTCs (*N* = 107,743) had more severe injuries (ICISS ≤ 0.85: 1.6% vs. 0.3%) compared to NTC discharges (*N* = 117,646) and lived in zipcodes where the nearest hospital was a DTC (41.3% vs. 11.5%). The predicted probability of discharge from DTC exceeded 0.77 irrespective of travel time when the nearest hospital was a DTC. However, when the nearest hospital was not DTC, the probability of discharge from DTC decreased as travel time to the nearest DTC increased, i.e., from 0.735 (95%CI: 0.689, 0.782) for < 12.5 min to 0.16 (95%CI: 0.128, 0.192) for > 38.8 min. This decrease was more pronounced for less-severe patients.

**Conclusions:**

A lower probability of discharge from a DTC corresponds to a higher probability of discharge from an NTC. When the nearest hospital was not DTC, the likelihood of NTC discharge increased with longer travel times to the nearest DTC. In Georgia, NTCs play a crucial role in the statewide trauma system by providing care to seriously injured patients when DTCs are geographically distant. These findings underscore the importance of NTCs in ensuring timely and accessible trauma care, particularly in regions where DTCs are not readily accessible.

**Supplementary Information:**

The online version contains supplementary material available at 10.1186/s40621-026-00665-6.

## Introduction

Treatment at Designated Trauma Centers (DTC) provides a survival advantage to severely injured trauma patients compared with treatments at hospitals that are not designated trauma centers, hereafter referred to as non-designated trauma centers (NTC) [1, 2]. However, NTCs also play a crucial role in trauma patient care by providing initial stabilization and care of trauma patients, particularly in areas where DTCs may not be readily accessible. Hospitals have to meet certain criteria to be DTCs, and depending on the criteria met, hospitals can be at different DTC levels, ranging from Level I to IV [3]. It is critically important that the trauma system triages patients to appropriate facilities, ensuring that those with more severe injuries are treated at higher-level trauma centers equipped to provide specialized care. Studies have shown that while direct transport of patients from the injury scene to a DTC is the most favorable triage, patients who are taken to emergency departments (ED) at NTCs before transfer to DTCs have survival benefits compared to those who are hospitalized at NTCs [4, 5]. However, the role of proximity to DTC vs. NTC remains understudied, even though 16% of the United States (US) population lacks geographic access to DTCs within 60 min of emergency medical services (EMS) transport [6].

In a state like Georgia, where the population is largely concentrated in one geographic region (one in three Georgians live in four counties around metro Atlanta), many Trauma Service Areas across the state with sparse populations do not meet the recommended number of DTCs based on the Need-Based Assessment of Trauma Systems guidelines [7, 8]. The Georgia Trauma System is composed of 10 regions, corresponding to 10 EMS regions for better coordination, with each region having its own Regional Trauma Advisor Committee [9, 10]. This committee develops trauma triage and destination guidelines for EMS. For example, Georgia Trauma Region 3 (that covers the Metro Atlanta Region, the most densely populated region in Georgia) follows the National Guideline for the Field Triage of Injured Patient criteria to triage trauma patients to either of their five adult DTCs, two pediatric DTCs, or NTCs [11]. However, the Georgia State Trauma System Consultation Report 2023 found that EMS agency medical directors can disregard these guidelines, leading to inconsistent triage and destination choices based on EMS judgment, proximity to DTCs/NTCs, or staying within their EMS zone [10]. In addition, though the majority of trauma patients are transported by EMS, non-EMS modes such as walk-ins/self-transport or brought in by family/bystanders are still prevalent [12]. Trauma triage at the injury site or trauma team activation at the hospital rarely occurs in non-EMS transport [13]. In such a scenario, proximity to the nearest hospital, access to personal vehicles, or patient preference might play a role in deciding the point of care rather than identifying the most appropriate hospital based on trauma triage [14]. The lack of DTCs in several geographic areas, coupled with variable triage and destination choices by EMS and non-EMS transports, may imply that trauma cases will be transported to NTCs, but the role of NTCs in such areas has not been studied.

Georgia is the 8th most populous state in the US, experiencing continued growth overall [8]. In areas where small populations are distributed across large geographical spaces with limited access to DTCs, NTCs may serve to fill the service gap by providing timely care for less severe trauma patients. The objective of this study is to identify factors such as geographical proximity to DTCs and trauma injury severity that influence the utilization of DTCs and NTCs for non-fatal trauma cases. We hypothesize that the severely injured patients are more likely to be treated and discharged from DTCs, whereas among less severely injured patients, the proximity to DTCs will affect whether they are likely to be treated or discharged from DTCs vs. NTCs. The distribution of trauma cases across different facility types, including DTCs and NTCs, highlights the role these centers play in providing care to trauma patients. In this study, we assess the role of NTCs in meeting trauma care needs for Georgia patients.

## Methods

### Study design

A retrospective cross-sectional study was conducted using Georgia’s de-identified non-fatal 2021 hospital discharge data from the Georgia Hospital Association (GHA). The study received Human Research Protection Program approval from the Institutional Review Board, University of Georgia.

### Trauma case identification

We followed the Georgia Trauma Registry 2021 guidelines to identify trauma patients, i.e., the patients who presented with at least one trauma-related International Classification of Diseases, Tenth Revision (ICD-10) code, and did not include patients with superficial wounds (see Supplementary Appendix A) [15]. We focus on adult patients to reduce heterogeneity due to the patient age group. While patient age is an important criterion in triaging [16, 17], factors like insurance, access to transportation, etc., also vary by patient age group. For example, Medicaid or private insurance would be the primary source of payment among children, children may also have access to transportation through guardians, including family members, whereas the elderly patient group will mostly have Medicare and may not have transportation access. Pediatric and elderly patients also have different field triage criteria set forth by the American College of Surgeons (ACS) [18]. In 2021, there were 728,446 non-duplicate, non-fatal trauma-related discharges from 600,082 unique patients, of which 60% (435,857discharges, 360,353 unique patients) were adult (18 to 64 years old) patients (Supplementary Appendix B).

To derive the analytic sample from the pool of adult trauma patients, we applied several additional criteria (Appendix B outlines the flowchart of the sample selection procedure). First, we included patients with either emergency, urgent, or trauma admit types (~ 76%), indicating an immediate care assessment by the medical staff at the admitting hospital. Prior studies [1, 19] investigating the state trauma system’s effectiveness has followed a similar approach, but excluded the urgent admit type, which we include (see robustness checks). We excluded elective (i.e., scheduled visits), newborn (i.e., incorrect category for adult patients), and those with missing admission type. Second, discharges with a point of origin for admission as missing (0.4%) were excluded. Third, we excluded patients with multiple hospital discharge records in the study year (20%) that are likely to be qualitatively different from those with single discharge records. To keep focused on patients with single discharge records, we also dropped 0.5% of discharges with “transfer from a hospital” as the point of origin for admission, i.e., our sample consists of patients that reached the facility that provided the care needed, consistent with past studies [1]. Finally, only trauma patients who are residents of Georgia and received care at DTCs in the Georgia Trauma Care system, which includes 5 DTCs in the state borderline with Florida, Tennessee, Alabama, and South Carolina were included in the study [7]. The final analytic sample consisted of single discharges from 225,389 unique adult trauma patients in 2021 (Supplementary Appendix B). No inclusion criteria based on patient type (ED vs. inpatient) or mode of transportation were applied. All hospital discharges were used, including ED discharges.

### Study measures

The main outcome of interest was trauma designation of discharging hospitals. Hospital discharges from any level DTCs (Level I to IV) were recoded as “1” while discharges from NTCs were coded as “0”. Information on hospitals’ addresses and trauma level designation (Level I through IV) for the study year was provided by GHA. The study sample had discharges from 35 DTCs and 102 NTCs representing 160 hospitals from Georgia (excluding Psychiatric/ Behavioral Health Hospitals) and 5 DTCs from neighboring states [20]. Supplementary Appendix C provides the map of Georgia with DTC locations.

The main predictors of interest were measures of geographic access to DTCs and patient injury severity. Two measures of access to DTCs were created using the Open Route Service API [21–24]: (1) an indicator to reflect whether the nearest hospital to the patient’s residence zipcode centroid (label point) was a DTC (1 if the nearest hospital is a DTC and 0 otherwise), and (2) road network time to reach the nearest DTC (may not be the nearest hospital) from the patient’s residence zipcode centroid. The API determines road network distance and driving time by constructing a graph-based representation of the road network, applying routing algorithms to identify the shortest path between points, and travel time estimates are based on road types and speed limits [21]. It was performed using the ArcGIS Pro “Create Drive-Time Areas” tool, which utilizes a time-neutral model based on historical average speeds rather than a single, specific time-of-day estimate. Road network time was used as a continuous measure and then categorized based on quartile splits: within 12.5 min, 12.5–22.8 min, 22.8–38.8 min, and more than 38.8 min. Though injury location would have provided more accurate measures of geographical access than the patient’s residence, the information was not available in the data set. However, past studies have found that most injuries occur closer to home [25, 26] and our analysis of 2019–2020 Georgia Trauma Registry Data found 63% of trauma incidents occurred at the patient’s residence zipcode (unpublished work). Thus, for all distance calculations, we assumed that trauma cases happened in the patient’s residence zipcode area and used it as a proxy for trauma incident location.

Injury severity was assessed using the ICD-10-based Injury Severity Score (ICISS). We adopted the ICISS, defined as the product of all survival risk ratios (SRR) for each of the patient’s injury ICD-10 codes, as defined in the literature [27, 28]. First, we estimated the SRR for injury ICD-10 codes using the National Inpatient Sample (NIS) 2018–2019. Second, the estimated SRR for every ICD-10 code from NIS 2018–2019 was matched to the same ICD-10 codes present in this study’s data. Finally, the SRR of all ICD-10 codes diagnosed for a patient was multiplied to obtain the ICISS for that patient. This is a standard approach to calculate ICISS that ranges from 0 to 1, with 0 indicating the most severe injury and 1 indicating the least severe injury [1]. The ICISS score was dichotomized, where cases with ICISS > 0.85 were considered low fatality threat (hereafter referred to as less-severe and coded “0”), and cases with ICISS ≤ 0.85 were considered high fatality threat (hereafter referred to as severe and coded “1”) based on prior studies [7, 19, 29].

To account for patient-specific and injury-specific factors that are associated with patient care and discharge from DTC, we included patient demographics (age, sex, race, ethnicity, primary payor, and rurality), patient health (Elixhauser Comorbidity Index risk for in-hospital mortality [ECI]), and injury indicators (intent, mechanism, and body region of injury) as covariates. Age was used as a continuous variable, whereas sex (male and female), ethnicity (Hispanic and non-Hispanic), race (White, Black, and Others if American Indian Alaskan Native, Asian, Native Hawaiian Pacific Islander, Other, and Refused), primary payer (Medicare, Medicaid, Other government, Department of Corrections, Private Health Insurance, Blue Cross/ Blue Shield, Managed Care (other), Self-pay, and Non-Payment), and rurality (rural and urban) were used as categorical measures. ECI risk for in-hospital mortality (refer to Healthcare Cost and Utilization Project for details) [30] was calculated to measure the comorbidity of the trauma patient instead of the commonly used Charlson Comorbidity Index (CCI), as recent studies report ECI to have better performance [31–34]. External cause-of-injury framework from the Centers for Disease Control and Prevention (CDC) was used to define the intent of injury (unintentional/accidental, self-harm, intentional/assault, and multiple intents), and mechanism of injury (cut/ pierce, drowning/submersion, fall, fire/burn, firearm, machinery, all transportation, nature/environmental, overexertion, poisoning, struck by/against, suffocation, other specified, unspecified, and multiple) [35]. CDC’s injury diagnosis framework was used to define the body region of injury (head, face, and neck, multiple regions, and all other regions) [36]. Supplementary Appendix D provides details on categories and the recoded values for the study measures. Discharge records with “unknown”, “refused”, “incorrect”, or “invalid” responses were retained for descriptive summaries but were dropped from regression analysis.

### Data analysis

We first described the patient characteristics, injury severity, and proximity indicators to the nearest hospital and nearest DTCs among the trauma patients who received care and discharge from a DTC vs. an NTC.

During regression model development, variable identification was conducted using a backward stepwise selection procedure on a complete case sample (200,719 in 2021) with the DTC vs. NTC discharge as a function of ICISS (continuous), and an interaction term to indicate whether the nearest hospital was a DTC and time to the nearest DTC, adjusted by the main effects of all covariates and a quadratic effect of age. Based on Akaike Information Criteria, the backward selection led to the exclusion of only the main effect of age.

Following backward selection, first, we implemented a logistic regression model (Model 1) to estimate the probability of DTC providing care and discharge (vs. NTC) and respective confidence intervals (CI) (see Supplementary Appendix E for details). Next, we conducted a secondary analysis (Model 2) that introduced a 3-way interaction term by adding ICISS (categorical) to the two-way interaction term in Model 1 to estimate the probability of DTC providing care and discharge (vs. NTC) by levels of injury severity, distance to nearest DTC, and trauma designation of nearest hospital (see Supplementary Appendix E for detail). Finally, a third logistic regression model based on Model 1 was implemented, where we replaced categorical road network time to the nearest DTC with a continuous measure to identify the inflection point, i.e., the odds of an event happening are equal to the odds of an event not happening.

### Robustness checks

We conducted several robustness checks. Studies have identified substance use as a major risk factor for morbidity and mortality from unintentional and intentional injuries [37–40]. However, substance-use-related ICD-10 codes were masked in our data; thus, such trauma patients could have been misclassified as less severe. Additionally, our sample included only non-fatal discharges (GHA’s hospital discharge data did not include fatal cases), and by design, led to the exclusion of severe trauma patients. Thus, we conducted a robustness check on Model 2, applying a less restrictive cutoff of 0.941 to categorize injury severity into severe (ICISS ≤ 0.941) and less-severe (ICISS > 0.941) injuries based on a prior study [41]. Second, we removed single records from January and December of the study year to ensure that trauma patients identified as single records were indeed single discharges and were not incorrectly included due to missing discharges in the preceding or following months in our data, i.e., December 2020 or January 2022. Third, among these records from February to November, we restricted the analytic sample to patients discharged to home, excluding those discharged to other facilities. Fourth, prior studies [1, 19] in Georgia had excluded the “urgent” admit type [42], i.e., patients who require immediate attention for the care and treatment of a physical or mental disorder. However, we decided to include such trauma patients in this study as there were 13,925 such trauma patients (out of 225,389 total patients), and among them, 6,130 (44%) received care and discharge from DTC, including 329 (5.4%) with inpatient stays. Thus, we conducted a robustness check by dropping cases with an urgent admit type from the sample identified in the third robustness check. R version 4.5.0 was used to conduct data analysis with the RStudio integrated development environment.

## Results

The socio-demographic distribution of trauma patients was similar across DTC and NTC with some notable differences: NTC discharges had a higher proportion of white patients, those enrolled in Blue Cross/Blue Shield, and injuries in other parts of body, whereas DTC discharges had higher proportion of those with no insurance (non-payment), living in urban areas, faced transportation-related injuries, and had multiple body regions of injury (Supplementary Appendix F).

A higher proportion of trauma patients discharged from DTCs were severe compared to those discharged from NTCs (1.6% vs. 0.3%) (Table 1). Similarly, compared to those discharged from NTCs, a higher proportion of patients living in zipcodes where the nearest hospital was a DTC were discharged from a DTC (41.3% vs. 11.5%), whereas a lower proportion of patients whose nearest hospital was not a DTC were discharged from a DTC (58.7% vs. 88.5%).

**Fig. 1:**
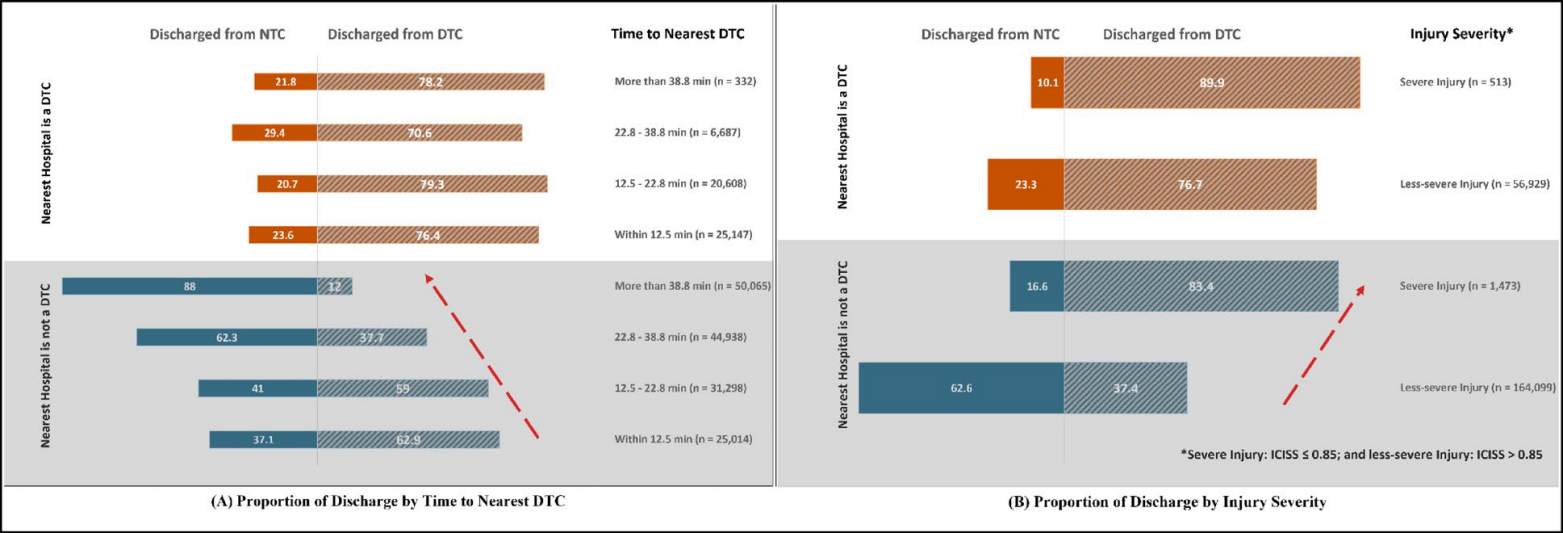
DTC vs. NTC Discharge when the nearest Hospital is a DTC vs. not a DTC: (**A**) Time to Nearest DTC and (**B**) Injury Severity

The majority (62.9%) of patients living in zipcodes where the nearest hospital was not DTC received care and were discharged from DTC when road network time to the nearest DTC was < 12.5 min (Fig. 1A, bottom left). This proportion decreased to 12.0% when road network time to the nearest DTC was > 38.8 min. However, the proportion of patients receiving care and discharged from a DTC remained consistent around 70.6% to 79.3% (Fig. 1A, top left) when the patient’s zipcode had the nearest hospital that was a DTC, irrespective of road network distance to the nearest DTC. Based on the patient’s ICISS score, severe patients were taken to DTCs regardless of whether the nearest hospital was a DTC. Notably, more than 80% of severe patients living in zipcodes where the nearest hospital was not DTC received care and were discharged from DTC (Fig. 1B).

**Fig. 2:**
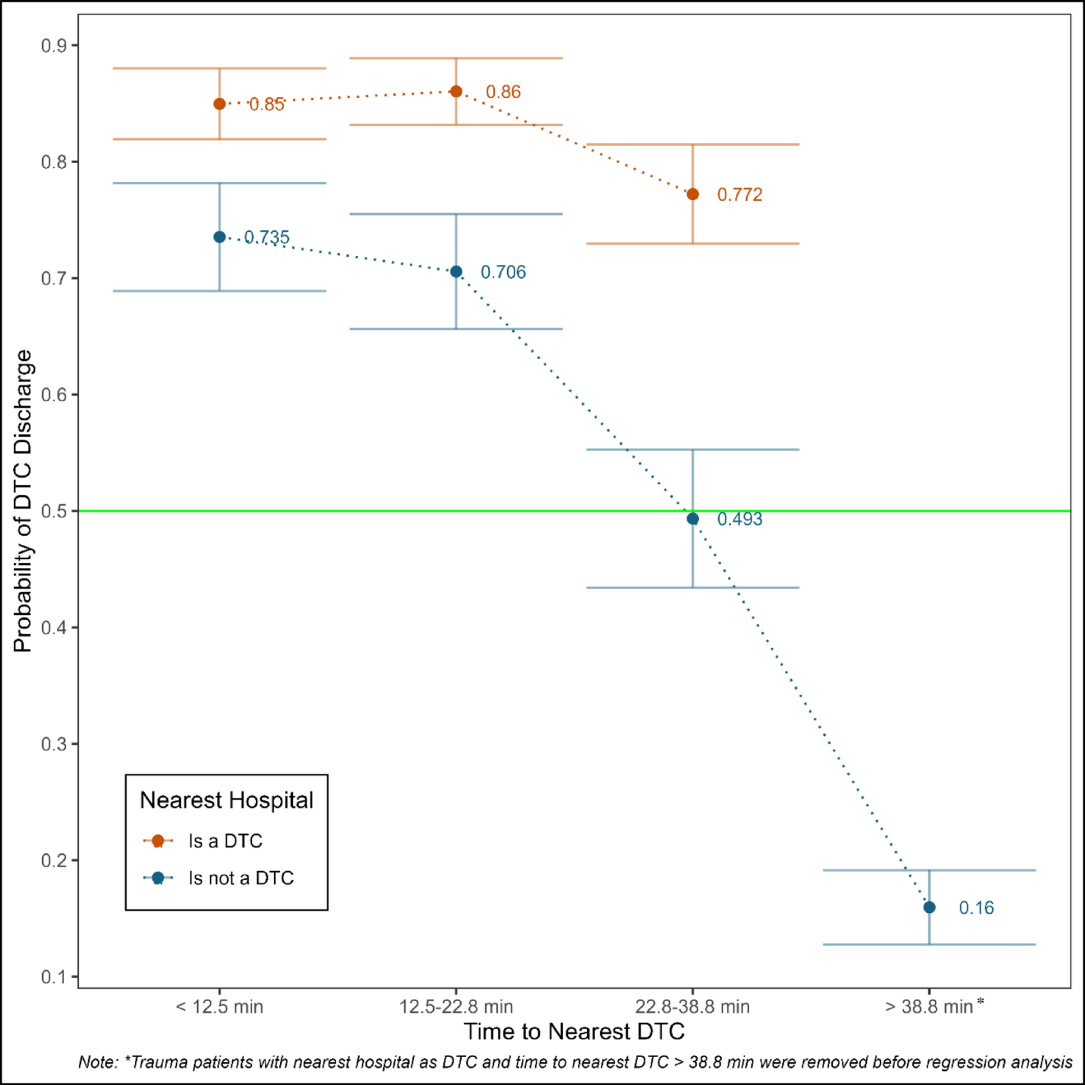
DTC Discharge Probabilities by Nearest Hospital’s Trauma Designation and Categorical Time to Nearest DTC

From the logistic regression, the probability of care and discharge from DTC was higher than 0.77, irrespective of the time to the nearest DTC when the nearest hospital to the patient’s zipcode was a DTC (Fig. 2). However, when the nearest hospital was not DTC, the probability decreased with an increase in time to the nearest DTC i.e., the probability of DTC discharge was 0.735 (95% CI: 0.689, 0.782) when time to the nearest DTC was < 12.5 min, whereas the probability of DTC discharge decreased to 0.16 (95% CI: 0.128, 0.192) when time to nearest DTC was > 38.8 min. Further, the gap between the probability of DTC discharge for the nearest hospital being a DTC vs. not a DTC widened significantly with increasing time (see Supplementary Appendix G Model 1 for the complete regression results).

**Fig. 3:**
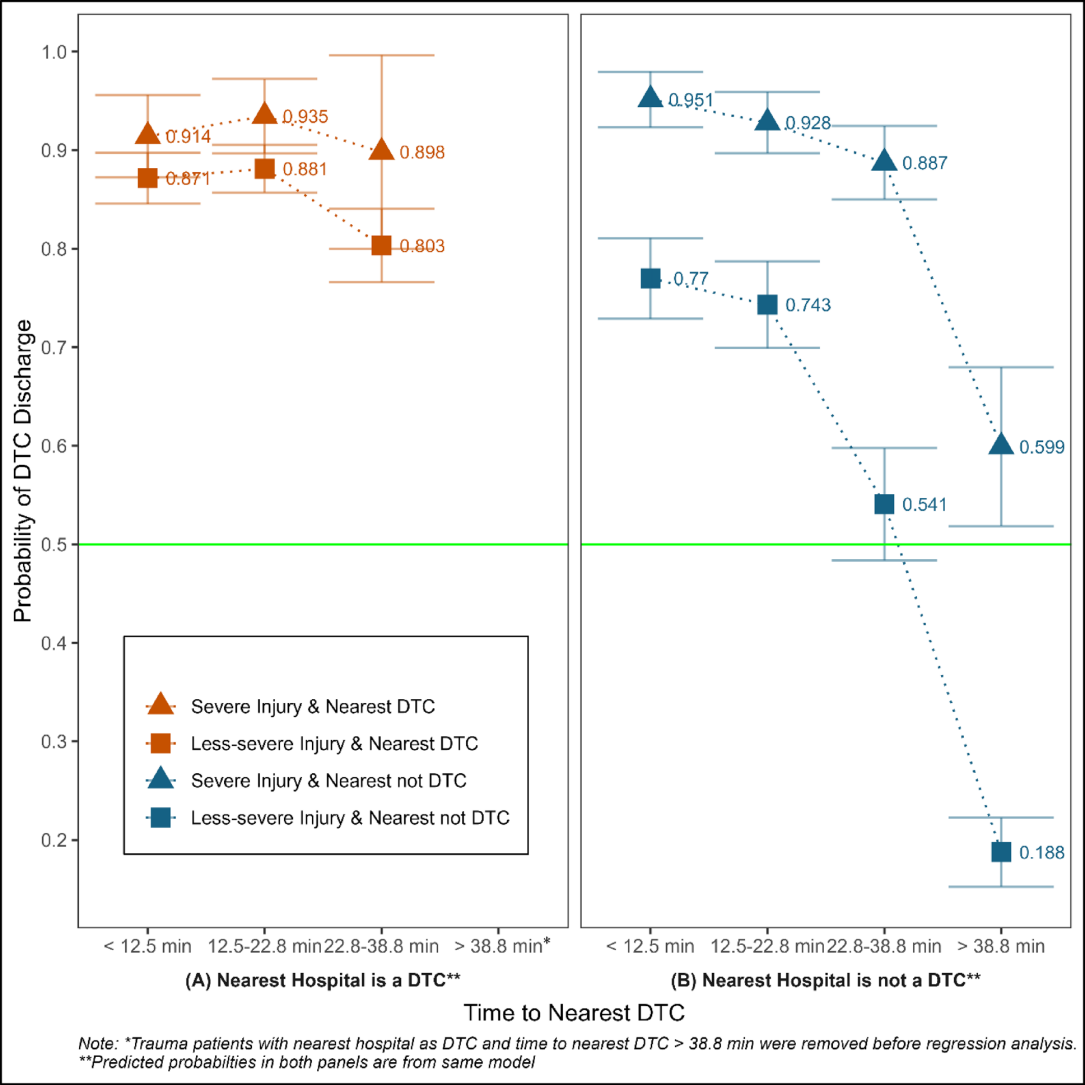
DTC Discharge Probabilities by Injury Severity, Nearest Hospital’s Trauma Designation, and Time to Nearest DTC

We also explored the differences in the probability of DTC discharge by injury severity. The probability of care and discharge from DTC was greater than 0.80 irrespective of the time to the nearest DTC when the nearest hospital to the patient’s zipcode was a DTC, with no statistical difference between severe and less-severe injuries (Fig. 3, left panel). However, when the nearest hospital was not DTC, the probability of DTC discharge significantly decreased with increasing time to the nearest DTC, with a more pronounced reduction in less-severe patients compared to severe patients (Fig. 3, right panel). For example, when the nearest hospital was not DTC, the probability of DTC discharge for severe patients decreased from 0.951 (95% CI: 0.923, 0.979) to 0.599 (95% CI: 0.518, 0.680) when the time to the nearest DTC increased from < 12.5 min to > 38.8 min. On the other hand, for less-severe patients, the probability decreased from 0.77 (95%CI: 0.729, 0.811) to 0.188 (95% CI: 0.153, 0.223) as time to the nearest DTC increased (see Supplementary Appendix G Model 2 for the complete regression results).

**Fig. 4:**
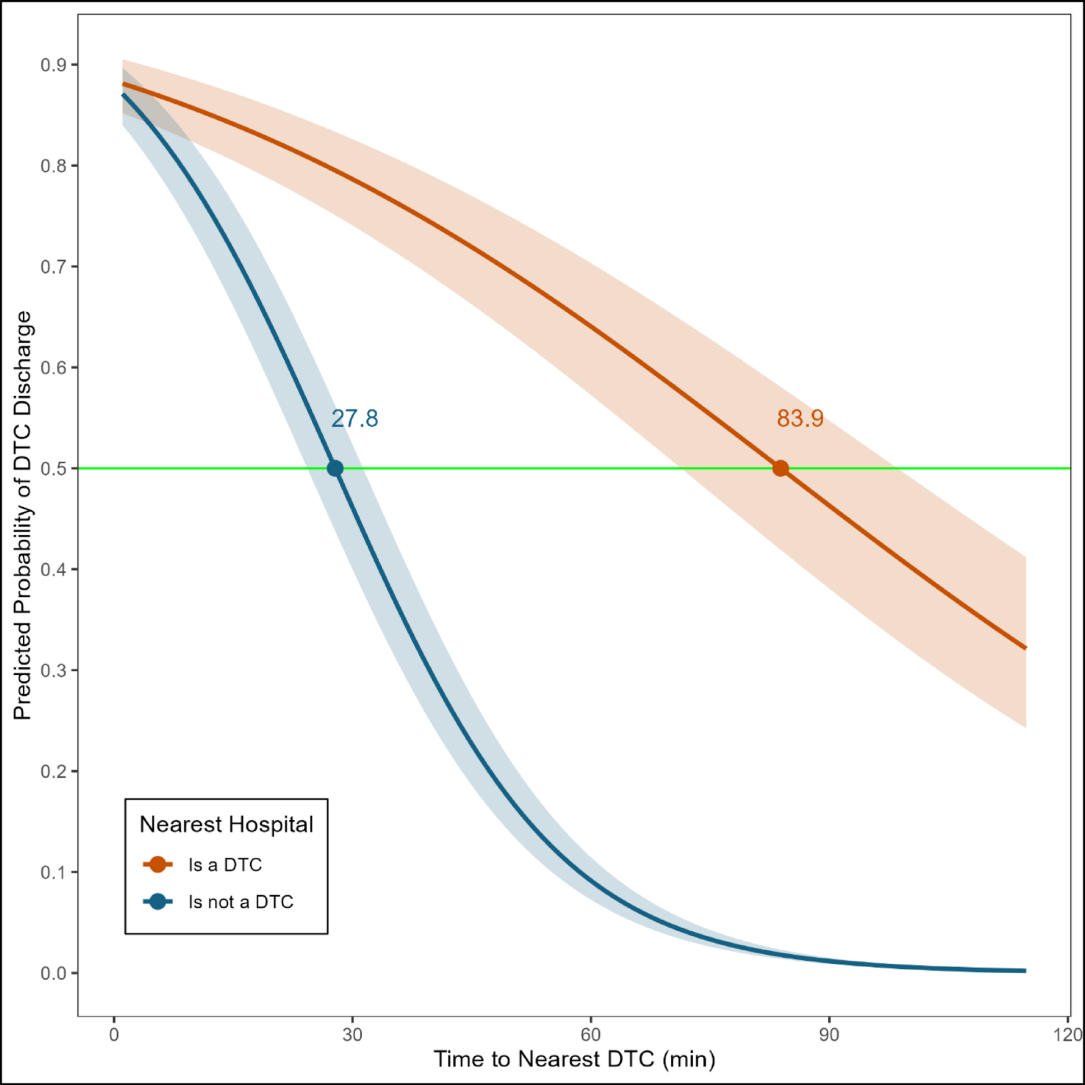
DTC Probabilities by Nearest Hospital’s Trauma Designation and Continuous Time to Nearest DTC

The inflection point for the time to the nearest DTC was 27.8 min when the nearest hospital was not a DTC and 83.9 min when the nearest hospital was a DTC (Fig. 4, see Supplementary Appendix G Model 3 for the complete regression results). It means that for a patient living in a zipcode where the nearest hospital is not a DTC, there is a higher probability (i.e., probability > 0.5) of being taken to a DTC for treatment if the time to the nearest DTC is less than about 28 min and if it is more than about 28 min, then such patients have a higher likelihood of being taken to an NTC. In contrast, when the nearest hospital is a DTC, the likelihood of DTC care and discharge remains higher so long as the time to the nearest DTC is less than about 84 min. Further, we observe a sharp decline in the probability of DTC discharge with an increase in time to the nearest DTC when the nearest hospital is not a DTC, such that it was less than 10% after 60 min of road network distance to the nearest DTC. However, the decline was relatively steady when the nearest hospital is DTC.

The findings remained robust, with similar predicted probabilities for receiving care and discharge from DTC when we used the ICISS cutoff of 0.941 (Supplementary Appendix H). Similarly, the results from Models 1 and 2 remained consistent across different sub-samples (Appendices I − K).

## Discussion

The study utilized non-fatal hospital discharge data from Georgia to identify the proximity to DTCs and injury severity as factors associated with care and discharge from DTCs vs. NTCs. The study findings suggest that distance to a DTC is an important barrier to receiving care from a DTC when the nearest hospital is not a DTC. Among these patients, the predicted probability of being discharged from a DTC ranges from 16% to 74%, with those living more than 38.8 min away experiencing the lowest probability (16%). The effect of distance is more pronounced among patients with less severe injuries. These findings highlight the importance of both the presence of DTC as the nearest hospital and the distance to the DTC from a patient’s residence in determining the choice of hospital type for trauma care.

The results have two key implications. First, the availability and quality of care at NTCs require greater attention. Our study found that NTCs provided care for up to 88.5% of trauma patients whose nearest hospital is not DTC, many of whom live in rural areas with significant geographic barriers. Further, our study suggests that patients living in areas where the nearest hospital is not DTC and the nearest DTC is farther than 28 min have a lower likelihood of being taken to DTC for treatment and discharge. However, if the patient was living in areas where the nearest hospital was a DTC, they have a higher likelihood of being taken to DTC as long as the nearest DTC is within 84 min. A national emergency department sample-based study also found that rural NTCs provided care for more than 50% of trauma patients, including 40% of the most severe injuries [43]. Jenkins et al. also report high-risk patients receiving treatment at NTCs with adequate resources rather than being transferred to DTCs [44]. However, trauma quality improvement efforts have largely focused on access to and processes within DTCs [45]. DTCs are better equipped to handle trauma cases as they provide a range of services, such as 24/7 organized trauma teams that include trauma surgeons and other key surgical specialties, rapid activation protocols, the ability to provide advanced trauma life support and stabilization, and emergency surgeries, which are not available at NTCs [46]. Thus, DTCs are more likely to provide quality care and have better patient outcomes [1]. NTCs in Georgia have minimal participation in the Regional Trauma Advisor Committee, which may impact coordination between centers to provide optimal care [10]. Expanding the trauma quality improvement efforts to include NTCs could help assess their role and performance in trauma care, particularly in underserved areas. For example, an evaluation of the Model Rural Trauma Project, a rural non-trauma center intervention in California (CA), focused on improving the management of major trauma at remote locations, found that NTCs were effective in providing care for trauma patients with the support of the trauma system [47]. Additionally, broader adoption of programs such as the Rural Trauma Team Development Course — which has been shown to reduce ED dwell time, expedite transfer decisions, and improve provider knowledge and confidence in rural hospitals—could help these centers better manage trauma patients despite resource constraints [48–50].

Second, the greater effect of distance on patients with less severe injuries suggests that triage practices may be functioning as intended by prioritizing severely injured patients for transport to DTCs. Evidence has found treatment and care for severely injured patients at DTCs to be beneficial [51]. The Georgia Trauma System is still in early stages; only in 2007 did the Senate Bill 60 establish the Georgia Trauma Care Network Commission to create a statewide trauma center network [52]. The higher probability of severely injured trauma patients being treated at the DTCs indicates maturity for the Georgia trauma system, however there are still a few areas of improvement as among patients with severe injuries whose nearest hospital was not DTC, the predicted probability of receiving care at a DTC declined by more than 30% points as distance increased from the first to the fourth quartile. Since DTCs provide the greatest benefit to severely injured patients and since the majority of trauma patients are transported by EMS [12], this finding indicates that the current triage system could benefit from enhanced EMS intervention to enable triage of patients with severe injury to DTCs, even from areas with limited access to DTCs, where distance may pose a significant barrier to appropriate care. For non-EMS transports, where proximity and patient preference might play significant role than EMS transport, inclusion of police/law enforcement [53] or education to bystanders [54] could be effective alternatives.

This study has a few limitations. First, geographical access indicators to DTCs are based on the patient’s residence zipcode and not the injury site zipcode. Additionally, the use of the zipcode centroid could result in misclassification for nearest hospital and road network time if patients reside in zipcode boundaries. Second, the study is limited to Georgia, and the findings may not be generalizable to other states. However, Georgia’s trauma system is highly concentrated in a few major counties near Atlanta, providing a relevant context for examining the effects of facility distribution and the access challenges faced by residents in non-metropolitan regions. Future studies may also include how the day or weekday versus weekend patterns impact trauma triage; such studies could utilize traffic heat map areas to find out alternate triage solutions during high traffic. Third, the study could not distinguish between patients arriving via EMS or self-presentation. If a trauma patient meets any one of the red or yellow criteria of the National guideline for the field triage of injured patients, EMS is more likely to present them to a DTC [18], whereas such rules are not applied to those self-presenting. However, national studies report higher utilization of EMS services, compared to non-EMS, by injured patients, with even higher utilization among moderate-to-severely injured patients [55, 56]. Additionally, evidence from the Georgia State Trauma System Consultation Report 2023 suggests that sometimes EMS might use their judgment or proximity to the nearest hospital instead of the national guideline to present a trauma patient to DTC or NTCs [10], which coincides with factors that an individual self-presenting to a hospital might consider. Future studies that merge hospital discharge data with either the Georgia Trauma Registry or Georgia Emergency Medical Services Information Systems data will help to address this issue. Fourth, the data were limited to trauma patients with a single discharge. Patients with fatal trauma and patients who are “transfer from a hospital” and repeat discharges are likely to be qualitatively different from those with single discharge records. For example, patients with fatal outcomes who are “transfer from a hospital” are more likely to be in severe condition and could have been taken to NTCs for survival care or transferred from one DTC to another DTC for a specialized procedure. The role NTCs play in survival care that we are unable to capture in this study suggests that our estimates might be biased towards the null and underestimating the actual patient volume at NTCs. Hospital selection by patients (outside EMS triage) with repeat discharge may be affected by factors outside proximity to trauma centers, such as perceptions of quality of care from prior discharges, which were outside the scope of this study. Fifth, we cannot differentiate EMS vs. non-EMS transport in our study. The role of proximity might be even higher among the non-EMS transport, where triage does not play a crucial role. Further study on modes of transportation is needed.

## Conclusion

The study shows that NTCs support the state trauma system by providing care for both less severe and, in some cases, more severe trauma patients in areas with limited access to DTCs. However, NTCs are not as properly equipped as DTCs to provide quality care to ensure survival and better patient outcomes. Efforts to include NTCs within the trauma system to ensure proper access to resources could play a critical role in ensuring timely trauma care, particularly when DTC resources are not readily available. The findings support the continuing education to NTCs around the evaluation and treatment of non-fatal, less severe trauma cases, such as through the ACS’s RTTDC. NTCs receiving severe trauma patients must also prioritize early transfer as per the base Advanced Trauma Life Support principles.

**Table 1:** Georgia Trauma Patients Characteristics Discharged by Designated Trauma Centers (DTC) and Non-Trauma Centers (NTC), 2021

Characteristics	Trauma Designation of Hospitals		
	DTC	NTC	Total
Number of discharges by hospital designation	107,743 [47.7%]	117,646 [52.3%]	225,389
**Injury Severity (ICISS score) (mean ± SD)**	0.996 ± 0.02	0.988 ± 0.05	0.992 ± 0.04
**Injury Severity**			
Less-severe (ICISS > 0.85)	105,057 [98.4%]	115,971 [99.7%]	221,028 [99.1%]
Severe (ICISS ≤ 0.85)	1690 [1.6%]	296 [0.3%]	1986 [0.9%]
Total	106,747	116,267	223,014
Missing	996	1379	2375
**Nearest Hospital is a**			
DTC for discharges	44,539 [41.3%]	13,473 [11.5%]	58,012 [25.7%]
Not a DTC for discharges	63,204 [58.7%]	104,173 [88.5%]	167,377 [74.3%]
Total	107,743	117,646	225,389
**Time to Nearest Hospital**,** min**	12.7 ± 8.6	13.2 ± 7.5	12.9 ± 8.1
**Time to Nearest DTC**,** min**	36.0 ± 21.5	18.2 ± 11.7	27.5 ± 19.6
**Time to Nearest DTC**			
Within 12.5 min	39,169 [36.4%]	17,179 [14.6%]	56,348 [25.0%]
12.5–22.8 min	37,798 [35.1%]	18,549 [15.8%]	56,347 [25.0%]
22.8–38.8 min	23,750 [22.0%]	32,597 [27.7%]	56,347 [25.0%]
> 38.8 min	7026 [6.5%]	49,321 [41.9%]	56,347 [25.0%]
Total	107,743	117,646	225,389

Categorical responses expressed as N [%] and continuous responses as mean ± SD

## Supplementary Information

Supplementary Material 1

## Data Availability

The data underlying this article cannot be shared publicly to maintain patient privacy.

## Abbreviations

ACS: American College of Surgeons
CDC: Centers for Disease Control and Prevention
DTC: Designated Trauma Center
ECI: Elixhauser Comorbidity Index risk for in-hospital mortality
ED: Emergency Department
EMS: Emergency Medical Services
GHA: Georgia Hospital Association
ICD-10: International Classification of Diseases, Tenth Revision
ICISS: International Classification of Disease-based Injury Severity Score
NIS: National Inpatient Sample
NTC: Non-Trauma Center
US: United States
