# Supplementary material for: Designated and non-designated trauma centers and trauma patients: a retrospective analysis of non-fatal trauma discharges in Georgia, 2021
Source: Inj Epidemiol. 2026 Feb 25;13:23. doi: 10.1186/s40621-026-00665-6 (PMC13041175; doi:10.1186/s40621-026-00665-6)
Supplement: Supplementary file 1 — Supplementary Material 1 [file 40621_2026_665_MOESM1_ESM.docx]

**Appendix A**

Inclusion and Exclusion Criteria to Identify Trauma Patients from Hospital Discharge Data Using 2021 Georgia Trauma Registry Trauma Patients Identification Criteria

**Inclusion Criteria:**

Patients with claims for the following ICD-10-CM codes

- S00-S99 w/ 7^th^ character modifiers of A, B, or C only
- T07; T14; T20-T28 with 7^th^ character A only or T30-T32; and T79.A1 – T79.A9 w/ 7^th^ character modifier A only

**Exclusion Criteria:**

Patients with claims for the following ICD-10-CM codes

- S00, S10, S20, S30, S40, S50, S60, S70, S80, S90
- Late effect codes w/ the 7^th^ character modifier of D through S

**Appendix B**

**GHA**

N: 884,963 (715,333)

**Duplicate records with same admission and discharge date**

n: 3,090

**Trauma Patients**

N: 728,446 (600,082)

**Inclusion of Patients with claims for following ICD-10 CM codes**

- S00-S99 with the 7^th^ character modifiers of A, B, or C only
- T07; T14; T20-T28 with the 7^th^ character A only or T30-T32; and T79.A1 – T79.A9 with the 7^th^ character modifier A only

**Exclusion of Patients with claims for following ICD-10 CM codes**

- S00, S10, S20, S30, S40, S50, S60, S70, S80, S90
- Late effect codes with the 7^th^ character modifier of D through S

**Non-Missing POA**

N: 305,534 (271,449)

**Multiple Discharge Records**

n: 60,841

**Non-duplicate Records**

N: 881,873 (715,298)

**Adult (age 18 – 64)**

N: 435,857 (360,353)

**Pediatric**: 143,024

**Elderly**: 149,546

**Missing Age:** 19

**Elective, Newborn, or Missing Admit Type**

n: 129,046

**Emergency, Urgent or Trauma Admit Type**

N: 306,811 (272,436)

**Missing Point of Origin (POA)**

n: 1,277

**Single Discharge Record**

N: 244,693 (244,693)

**No Intermediate Stop**

N: 243,442 (244,693)

**Transfer from Hospital as POA**

n: 1,251

**Trauma Patients Outside of GA**

n: 18,053

**Final Analytical Sample**

N: 225,389 (225,389)

**Figure B.1: Study Sample Selection Procedure [numbers presented as “discharge record count (unique patient count)”]**

**Appendix C**


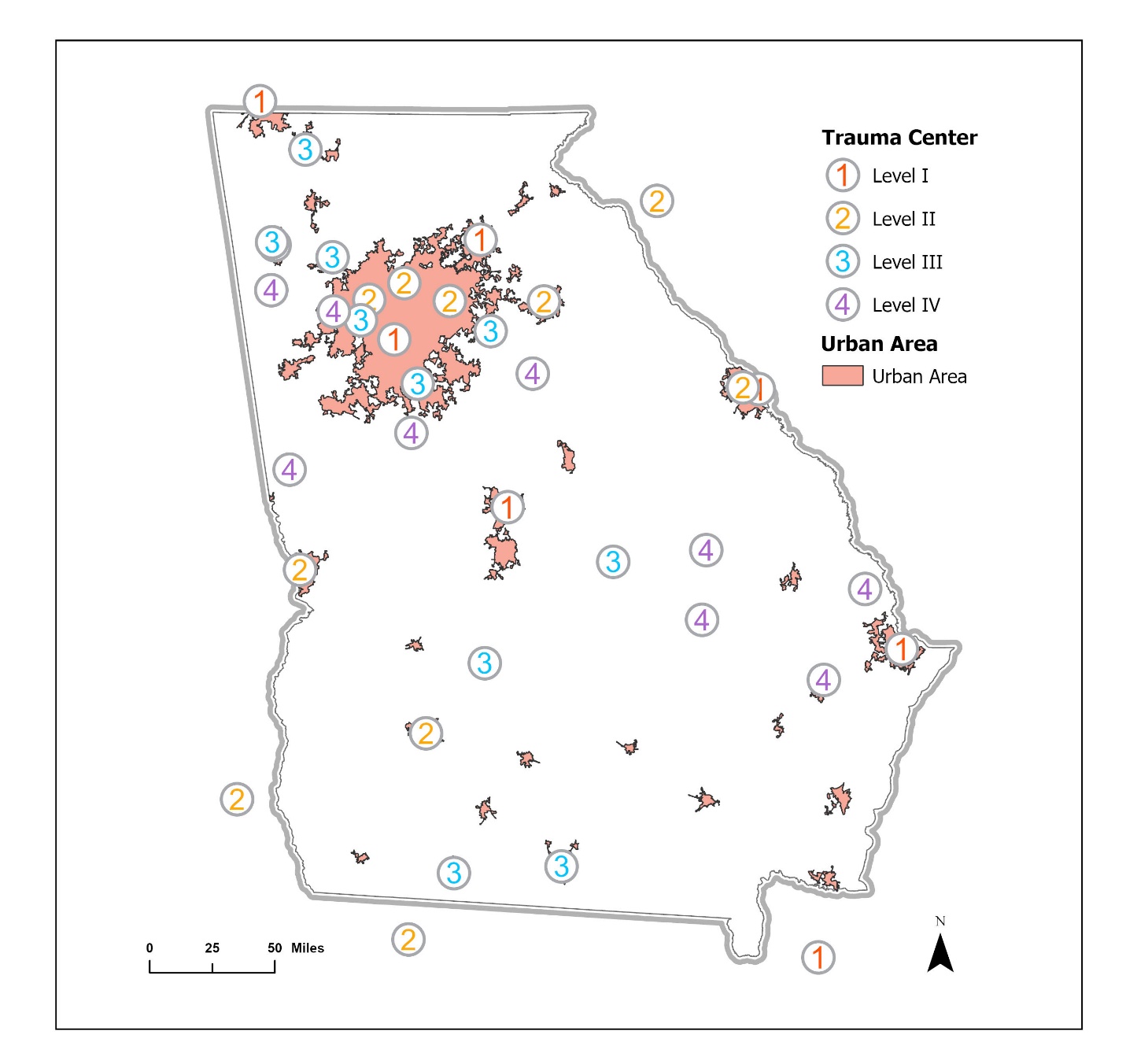


**Figure C.1: Map of Georgia with DTC Location and Urban Area**

**Appendix D**

**Table D.1: Description of Study Measures**

| **Study Measure** | **Short Description** | **Response Options** | **Recode, if any** |
| --- | --- | --- | --- |
| Trauma Center Designation | Trauma level status of the facility where the patient received care | - Trauma Level I - Trauma Level II - Trauma Level III - Trauma Level IV - No Trauma Designation | - 0: No Trauma Designation - 1: Trauma Level I – IV |
| Nearest Hospital Trauma Designation | Trauma Designation of the Nearest Hospital from the patient’s zip code | - 0: Nearest hospital is not a DTC - 1: Nearest hospital is a DTC |  |
| Time to Nearest Hospital | Road network time from the patient’s zip code to the nearest Hospital | Continuous, in seconds |  |
| Time to Nearest Trauma Center | Road network time from the patient’s zip code to the nearest DTC | Continuous, in seconds | Categories based on quartile split: Within 12.5 min; 12.5 – 22.8 min; 22.8 – 38.8 min; and > 38.8 min |
| International classification of disease-based injury severity score (ICISS) | ICISS was defined as the product of all survival risk ratios (i.e., cumulative approach) for each of the patient’s injury ICD-10 codes (equation below). The survival risk ratios for each ICD-10 code were estimated using the National Inpatient Sample (NIS) from 2018 and 2019 and then applied to the hospital discharge data for this study to estimate the ICISS.  ICISS = $P_{{SurvInj}_{1}}\times P_{{SurvInj}_{2}}\times\ldots\times P_{{SurvInj}_{n}}$  where, $P_{{SurvInj}_{n}}$is the survival for the injury type n (a specific ICD-10 code) from the NIS 2018 and 2019 data. | Ranges from 0 to 1 where “0” represents the most severe injury, while 1 indicates the least severe injury | - Severe: denotes high fatality threat, i.e., if ICISS ≤ 0.85 - Less severe: denotes low fatality threat, i.e., if ICISS > 0.85 |
| Age | Age | Continuous |  |
| Sex | Patient’s sex | - Male - Female - Unknown | - Male - Female   Note: Unknown “sex” removed from sample |
| Race | Patient’s Race | - White - Black - American Indian Alaskan Native - Asian - Native Hawaiian Pacific Islander - Other - Patient Refused - Invalid Response - Missing | - White - Black - Other: American Indian Alaskan Native, Asian, Native Hawaiian, Pacific Islander, Other, or Patient Refused |
| Ethnicity | Patient’s Ethnicity | - Hispanic/Latino - Not Hispanic/Latino - Invalid Response | - Hispanic/Latino - Not Hispanic/Latino |
| Primary Payor | Primary insurance payor type | - Medicare - Medicaid - Other government - Department of Corrections - Private Health Insurance - Blue Cross/ Blue Shield - Managed Care, other - Self-pay - Non-Payment |  |
| Rurality | Urban area boundaries in GA are derived from the US TIGER dataset*. The classification of a ZIP code as urban or rural is determined by whether its centroid falls within an urban boundary. | - Urban: If the zip code’s centroid falls within an urban boundary - Rural: If the zip code’s centroid does not fall within an urban boundary |  |
| Elixhauser Comorbidity Index risk for in-hospital mortality (ECI) | Following the guidelines from the Health Care Utilization Project (HCUP), ECI risk for in-hospital mortality was calculated to measure the comorbidity of the trauma patient. | Continuous | - Low Risk: if ECI ≤ 0 - At Risk: if ECI > 0 |
| Intent of Injury | Based on the CDC’s external cause-of-injury framework**, intent of injury was defined as whether the injury was inflicted purposefully and by whom.  It was created based on the ICD-10 code by the authors. | - Unintentional/accidental - Self-harm - Intentional/assault | - Unintentional/accidental: if only unintentional/accidental was the intent - Self-harm: if only self-harm was the intent - Intentional/assault: if only intentional/assault was the intent - Multiple intent: if more than one type of intent for injury |
| Mechanism of Injury | Based on the CDC’s external cause-of-injury framework**, the mechanism of injury was defined as the vector that transfers energy to the body | - Cut/ pierce - Drowning/submersion - Fall - Fire/burn - Firearm - Machinery - All transportation - Nature/environmental - Overexertion - Poisoning - Struck by/against - Suffocation - Other Specified - Unspecified | - Cut/ pierce: if only cut/pierce was the mechanism - Drowning/submersion: if only drowning/submersion was the mechanism - Fall: if only fall was the mechanism - Fire/burn: if only fire/burn was the mechanism - Firearm: if only the firearm was the mechanism - Machinery: if only machinery was the mechanism - All transportation: if only transportation was the mechanism - Nature/environmental: if only nature/environment was the mechanism - Overexertion: if only overexertion was the mechanism - Poisoning: if only poisoning was the mechanism - Struck by/against: if only struck by/against was the mechanism - Suffocation: if only suffocation was the mechanism - Other Specified: if only other specified was the mechanism - Unspecified: if only unspecified was the mechanism - Multiple: if more than one type of mechanism of injury |
| Body Region of Injury | Based on the CDC’s injury diagnosis framework***, the body region of injury was defined as the body parts affected during the injury | - Head, face, and neck - Trunk - Shoulder and forearm - Wrist, hand, and fingers - Lower limb - Ankle and foot - Other internal organs - Multiple regions | - Head, face, and neck: if only TBI, other head, neck, and head and neck, other was the body region of injury - Multiple regions: if more than one type of body region of injury, or had multiple regions as body regions of injury - All Other Regions: if the body region of injury was one of the remaining sites |

Note: *US Tiger Dataset available at <https://www.census.gov/geographies/mapping-files/time-series/geo/tiger-line-file.2021.html#list-tab-790442341>; **CDC’s external cause-of-injury framework is available at <https://www.cdc.gov/nchs/data/nhsr/nhsr136-508.pdf>; ***CDC’s injury diagnosis framework is available at <https://www.cdc.gov/nchs/data/nhsr/nhsr150-508.pdf>

**Appendix E**

**Detailed Description of Model 1**

Following model 1 was implemented to estimate the probability of DTC providing care and discharge (vs. NTC) and respective confidence intervals (CI):

| logit(P(Discharge from DTC)) = | $\beta_{0}$ + $\beta_{1}\left( ICISS \right)$ + $\beta_{2}(Nearest)$ + $\beta_{3a}{(Time)}_{a}$ + $\beta_{4b}{(Nearest\times Time)}_{b}$ + $\varphi X_{i}$ + $\varepsilon$ **(1)** |
| --- | --- |

where *ICISS* is the ICD-10-based Injury Severity Score (continuous), *Nearest* is the indicator variable that takes on the value “1” if the nearest hospital to the patient's zipcode was a DTC, “0” otherwise, *Time_a_* is the road network time to the nearest DTC, where “a” $\in$ (12.5 – 22.8 min, 22.8 – 38.8 min, and > 38.8 min) and reference “≤ 12.5 min”, *(Nearest × Time)* is the categorical-by-categorical interaction term of an indicator if the nearest hospital was DTC and road network time to the nearest DTC, *X_i_* is the set of covariates where “i” $\in$ (quadratic effect of age, and main effects of sex, race, ethnicity, primary payor, ECI, injury intent, mechanism of injury, and body region of injury), and $\varepsilon$ is the error term.

Model 1 estimated seven such predicted probabilities for each predictor level, i.e., 4 levels of road network time to the nearest DTC when the nearest hospital was NTC, and 3 levels of road network time to the nearest DTC when the nearest hospital was DTC. Before regression analysis, trauma patients who were living in areas with the nearest hospital as DTC and time to nearest DTC more than 38.8 mins (main predictors of interest) were removed due to low sample size between comparator groups i.e., <10 severe trauma patients vs. 356 less-severe trauma patients for that group, thus we had only 3 levels of road network time when nearest hospital was a DTC unlike 4 levels when nearest hospital was not a DTC.

**Detailed Description of Model 2**

Model 2 introduced a 3-way interaction term by adding ICISS (categorical) to the two-way interaction term in Model 1 to estimate the probability of DTC providing care and discharge (vs. NTC) by levels of injury severity, distance to nearest DTC, and trauma designation of nearest hospital. There are 14 such levels in Model 2, i.e., 2 levels of ICISS category × 4 levels of road network time to the nearest DTC when the nearest hospital was NTC, and 2 levels of ICISS category × 3 levels of road network time to the nearest DTC when the nearest hospital was DTC.

| logit(P(Discharge from DTC)) = | $\beta_{0}$ + $\beta_{1}\left( {ICISS}_{cat} \right)$ + $\beta_{2}(Nearest)$ + $\beta_{3a}{(Time)}_{a}$ + $\beta_{4b}{({ICISS}_{cat}\times Nearest\times Time)}_{b}$ + $\varphi X_{i}$ + $\varepsilon$ **(2)** |
| --- | --- |

where *ICISS_cat_* is a dichotomous ICISS score that takes on the value “1” if the injury was severe (i.e., ICISS ≤ 0.85), “0” if less-severe (i.e., ICISS > 0.85), and ${({ICISS}_{cat}\times Nearest\times Time)}_{b}$ is the three-way interaction term between ICISS (categorical), an indicator of whether the nearest hospital was DTC, and road network time to the nearest DTC.

**Detailed Description of Model 3**

Model 3 was implemented, based on Model 1, where we replaced the categorical road network time to the nearest DTC with a continuous measure to identify the inflection point. Such a point occurs when the predicted probability of receiving care and discharge from a DTC is 0.50, i.e., no difference in predicted probabilities of receiving care and discharge from a DTC vs. NTC

| logit(P(Discharge from DTC)) = | $\beta_{0}$ + $\beta_{1}\left( ICISS \right)$ + $\beta_{2}(Nearest)$ + $\beta_{3}Time$ + $\beta_{4}(Nearest\times Time)$ + $\varphi X_{i}$ + $\varepsilon$ **(3)** |
| --- | --- |

where *Time* is the road network time to the nearest DTC measured on a continuous scale,

The logit of the probability of receiving care and discharge from DTC is 0 when the predicted probability is 0.5. Thus, after replacing the value of “*logit(P(Discharge from DTC))*” with 0 and solving for “*Time*”, Model 3 can be re-written as Equation (i) when the nearest hospital is not a DTC i.e., value for “*Nearest*” is 0, and Equation (ii) when the nearest hospital is a DTC i.e., value for “*Nearest*” is 1. The value of “*Time*” obtained from Equations (i) and (ii) provides the inflection point where the predicted probability of receiving care and discharge from DTC is 0.5 when the nearest hospital is not a DTC and when it is a DTC, respectively, averaged over other characteristics.

| ${Time}_{not a DTC}$ | = | $-\left( \frac{\beta_{0} + \beta_{1}\left( ICISS \right) + \varphi X_{i}}{\beta_{3}} \right)$ (i) |
| --- | --- | --- |
| ${Time}_{DTC}$ | = | $-\left( \frac{\beta_{0} + \beta_{1}\left( ICISS \right) + \beta_{2}+ \varphi X_{i}}{\beta_{3} + \beta_{4}} \right)$ (ii) |

**Appendix F**

**Table E.1: Patient Characteristics by Discharging Hospital’s Trauma Designation, 2021**

| **Characteristics** | **Trauma Designation** | | | |
| --- | --- | --- | --- | --- |
|  | **DTC, 107,743 [47.7%]** | | **NTC, 117,646 [52.3%]** | |
|  | **N** | **%** | **N** | **%** |
| **Age (mean ± SD)** | 38.3 ± 13.4 |  | 39.0 ± 13.5 |  |
| **Sex** |  |  |  |  |
| Female | 49,829 | 46.3% | 59,121 | 50.3% |
| Male | 57,816 | 53.7% | 58,518 | 49.7% |
| Total | 107,645 |  | 117,639 |  |
| Unknown | 98 |  | <10 |  |
| **Ethnicity** |  |  |  |  |
| Hispanic | 7,736 | 7.2% | 4,834 | 4.1% |
| Non-Hispanic | 100,005 | 92.8% | 112,768 | 95.9% |
| Total | 107,741 |  | 117,602 |  |
| Invalid Reported | <10 |  | 44 |  |
| **Race** |  |  |  |  |
| White | 46,721 | 43.4% | 59,876 | 50.9% |
| Black | 49,569 | 46.0% | 49,306 | 41.9% |
| American Indian/Native Alaskan | 230 | 0.2% | 887 | 0.8% |
| Asian | 996 | 0.9% | 1,348 | 1.1% |
| Native Hawaiian/Pacific Islander | 157 | 0.1% | 298 | 0.3% |
| Other | 8,840 | 8.2% | 4,289 | 3.6% |
| Refused | 1,191 | 1.1% | 1,552 | 1.3% |
| Total | 107,704 |  | 117,556 |  |
| Not reported | - |  | 21 |  |
| Invalid Reported | 25 |  | <10 |  |
| Missing | 14 |  | 65 |  |
| **Primary Payor** |  |  |  |  |
| Medicare | 5,903 | 5.5% | 7,207 | 6.2% |
| Medicaid | 15,252 | 14.2% | 16,147 | 14.0% |
| Other Government | 2,174 | 2.0% | 3,552 | 3.1% |
| Department of Corrections | 675 | 0.6% | 629 | 0.5% |
| Private Health Insurance | 18,652 | 17.3% | 18,973 | 16.4% |
| Blue Cross/Blue Shield | 9,238 | 8.6% | 15,746 | 13.6% |
| Managed Care, other | 4,544 | 4.2% | 5,734 | 5.0% |
| Self-pay | 23,132 | 21.5% | 29,400 | 25.4% |
| Non-Payment | 10,492 | 9.7% | 2,504 | 2.2% |
| Others | 17,622 | 16.4% | 15,650 | 13.5% |
| Total | 107,684 |  | 115,542 |  |
| Missing | 59 |  | 2,104 |  |
| **Rurality** |  |  |  |  |
| Rural | 24,505 | 22.7% | 44,115 | 37.5% |
| Urban | 83,238 | 77.3% | 73,531 | 62.5% |
| Total | 107,743 |  | 117,646 |  |
| **ECI (mean ± SD)** | 0.32 ± 3.93 |  | 0.11 ± 3.93 |  |
| **ECI** |  |  |  |  |
| ≤ 0 | 94,860 | 88.0% | 108,846 | 92.5% |
| > 0 | 12,883 | 12.0% | 8,800 | 7.5% |
| Total | 107,743 |  | 117,646 |  |
| **Intent of Injury** |  |  |  |  |
| Unintentional/accidental | 90,313 | 92.3% | 104,728 | 95.2% |
| Self-harm | 904 | 0.9% | 643 | 0.6% |
| Intentional/assault | 6,273 | 6.4% | 4,452 | 4.0% |
| Multiple intents | 358 | 0.4% | 194 | 0.2% |
| Total | 97,848 |  | 110,017 |  |
| Missing | 9,895 |  | 7,629 |  |
| **Mechanism of Injury** |  |  |  |  |
| Cut/ pierce | 10,151 | 10.5% | 13,947 | 12.7% |
| Fall | 18,132 | 18.7% | 22,770 | 20.8% |
| Fire/burn | 1,673 | 1.7% | 1,740 | 1.6% |
| Firearm | 1,673 | 1.7% | 397 | 0.4% |
| Machinery | 71 | 0.1% | 81 | 0.1% |
| All transportation | 26,813 | 27.7% | 21,688 | 19.8% |
| Nature/Environmental | 2,379 | 2.5% | 3,444 | 3.1% |
| Overexertion | 6,507 | 6.7% | 11,123 | 10.2% |
| Poisoning | 209 | 0.2% | 233 | 0.2% |
| Struck by/against | 11,097 | 11.5% | 12,992 | 11.9% |
| Other Specified | 2,070 | 2.1% | 2,399 | 2.2% |
| Unspecified | 10,755 | 11.1% | 14,612 | 13.4% |
| Multiple | 5,265 | 5.4% | 3,995 | 3.7% |
| Total | 96,795 |  | 109,421 |  |
| Drowning/submersion | 10 |  | <10 |  |
| Suffocation | <10 |  | <10 |  |
| Missing | 10,935 |  | 8,220 |  |
| **Body Region of Injury** |  |  |  |  |
| All Other Regions | 66,961 | 62.2% | 83,349 | 70.9% |
| Head, face, and neck | 17,943 | 16.7% | 17,436 | 14.8% |
| Multiple regions | 22,825 | 21.2% | 16,844 | 14.3% |
| Total | 107,729 |  | 117,629 |  |
| Missing | 14 |  | 17 |  |

Note: Categorical responses expressed as N [%] and continuous responses as mean ± SD; and p-values from the two-sample independent test of means and proportion for continuous and categorical responses, respectively.

**Appendix G**

**Table G.1: Odds of Discharge from DTC vs. NTC from the logistic regression models**

| **Study Measures** | **Odds Ratio of DTC vs. NTC Discharge** | | | | | |
| --- | --- | --- | --- | --- | --- | --- |
|  | **Model 1** | | **Model 2** | | **Model 3** | |
| **ICISS, continuous** | 0.00 [0.00, 0.00] |  | | 0.00 [0.00, 0.00] | |  |
| **ICISS** |  |  | |  | |  |
| (ref. Less-severe (ICISS > 0.85)) |  |  |  |  |  |  |
| Severe (ICISS ≤ 0.85) |  | 5.84 [3.46, 10.8] | |  | |  |
| **Nearest Hospital is a** |  |  | |  | |  |
| (ref. Not a DTC) |  |  |  |  |  |  |
| DTC for discharges | 2.03 [1.95, 2.11] | 2.02 [1.94, 2.11] | | 1.04 [0.99, 1.10] | |  |
| **Time to Nearest DTC** |  |  | |  | |  |
| (ref. Within 12.5 min) |  |  |  |  |  |  |
| 12.5 – 22.8 min | 0.86 [0.83, 0.89] | 0.86 [0.83, 0.89] | |  | |  |
| 22.8 – 38.8 min | 0.35 [0.33, 0.36] | 0.35 [0.33, 0.36] | |  | |  |
| > 38.8 min | 0.06 [0.06, 0.07] | 0.06 [0.06, 0.07] | |  | |  |
| **Time to Nearest DTC, continuous** |  |  | | 0.93 [0.93, 0.93] | |  |
| **Nearest Hospital × Time to Nearest DTC** |  |  | |  | |  |
| (ref. DTC for discharges & Within 12.5 min) |  |  |  |  |  |  |
| DTC for discharges & 12.5 – 22.8 min | 1.26 [1.19, 1.33] | 1.26 [1.19, 1.33] | |  | |  |
| DTC for discharges & 22.8 – 38.8 min | 1.71 [1.59, 1.83] | 1.70 [1.59, 1.83] | |  | |  |
| **Nearest Hospital × Time to Nearest DTC, continuous** |  |  | | 1.04 [1.04, 1.05] | |  |
| **ICISS × Nearest Hospital** |  |  | |  | |  |
| (ref. Severe & not a DTC for discharges) |  |  |  |  |  |  |
| Severe & DTC for discharges |  | 0.26 [0.12, 0.55] | |  | |  |
| **ICISS × Time to Nearest DTC** |  |  | |  | |  |
| (ref. Severe & Within 12.5 min) |  |  |  |  |  |  |
| Severe & 12.5 – 22.8 min |  | 0.76 [0.36, 1.49] | |  | |  |
| Severe & 22.8 – 38.8 min |  | 1.14 [0.57, 2.11] | |  | |  |
| Severe & > 38.8 min |  | 1.10 [0.57, 1.98] | |  | |  |
| **ICISS × Nearest Hospital × Time to Nearest DTC** |  |  | |  | |  |
| (ref. Severe & not a DTC for discharges & Within 12.5 min) |  |  |  |  |  |  |
| Severe & DTC for discharges & 12.5 – 22.8 min |  | 1.20 [0.58, 4.57] | |  | |  |
| Severe & DTC for discharges & 22.8 – 38.8 min |  | 1.99 [0.34, 5.06] | |  | |  |
| **Age^2** | 1.00 [0.99, 1.00] | 1.00 [1.00, 1.00] | | 1.00 [1.00, 1.00] | |  |
| **Sex** |  |  | |  | |  |
| (ref. Female) |  |  |  |  |  |  |
| Male | 1.17 [1.15, 1.20] | 1.20 [1.17, 1.22] | | 1.17 [1.14, 1.20] | |  |
| **Ethnicity** |  |  | |  | |  |
| (ref. Hispanic) |  |  |  |  |  |  |
| Non-Hispanic | 0.76 [0.72, 0.80] | 0.76 [0.72, 0.80] | | 0.78 [0.74, 0.82] | |  |
| **Race** |  |  | |  | |  |
| (ref. White) |  |  |  |  |  |  |
| Black | 0.89 [0.87, 0.92] | 0.89 [0.87, 0.91] | | 0.88 [0.85, 0.90] | |  |
| Other | 1.09 [1.05, 1.14] | 1.10 [1.05, 1.15] | | 1.09 [1.04, 1.14] | |  |
| **Primary Payor** |  |  | |  | |  |
| (ref. Medicare) |  |  |  |  |  |  |
| Medicaid | 1.09 [1.03, 1.15] | 1.09 [1.04, 1.15] | | 1.09 [1.03, 1.15] | |  |
| Other Government | 0.60 [0.56, 0.66] | 0.60 [0.55, 0.65] | | 0.61 [0.56, 0.66] | |  |
| Department of Corrections | 1.03 [0.88, 1.20] | 1.03 [0.89, 1.21] | | 1.11 [0.94, 1.30] | |  |
| Private Health Insurance | 0.99 [0.94, 1.04] | 0.99 [0.94, 1.04] | | 1.00 [0.95, 1.06] | |  |
| Blue Cross/Blue Shield | 0.72 [0.68, 0.76] | 0.71 [0.67, 0.75] | | 0.72 [0.68, 0.76] | |  |
| Managed Care, other | 0.56 [0.52, 0.60] | 0.56 [0.52, 0.60] | | 0.56 [0.52, 0.59] | |  |
| Self-pay | 0.75 [0.71, 0.79] | 0.75 [0.71, 0.78] | | 0.74 [0.70, 0.78] | |  |
| Non-Payment | 3.64 [3.40, 3.90] | 3.63 [3.39, 3.89] | | 3.85 [3.59, 4.12] | |  |
| Others | 1.24 [1.18, 1.31] | 1.24 [1.18, 1.31] | | 1.28 [1.21, 1.35] | |  |
| **Rurality** |  |  | |  | |  |
| (ref. rural) |  |  |  |  |  |  |
| Urban | 0.70 [0.68, 0.72] | 0.71 [0.69, 0.73] | | 0.63 [0.61, 0.64] | |  |
| **ECI** | 1.00 [1.00, 1.00] | 1.00 [1.00, 1.00] | | 1.00 [1.00, 1.00] | |  |
| **Intent of Injury** |  |  | |  | |  |
| (ref. Unintentional/accidental) |  |  |  |  |  |  |
| Self-harm | 1.62 [1.43, 1.84] | 1.76 [1.55, 2.00] | | 1.62 [1.42, 1.84] | |  |
| Intentional/assault | 1.58 [1.50, 1.67] | 1.63 [1.54, 1.72] | | 1.56 [1.47, 1.65] | |  |
| Multiple intents | 1.47 [1.19, 1.83] | 1.55 [1.26, 1.92] | | 1.43 [1.15, 1.77] | |  |
| **Mechanism of Injury** |  |  | |  | |  |
| (ref. cut/pierce) |  |  |  |  |  |  |
| Fall | 1.05 [1.00, 1.09] | 1.08 [1.04, 1.13] | | 1.05 [1.00, 1.09] | |  |
| Fire/burn | 1.29 [1.18, 1.41] | 1.34 [1.23, 1.46] | | 1.30 [1.19, 1.42] | |  |
| Firearm | 5.28 [4.61, 6.06] | 6.23 [5.45, 7.13] | | 5.40 [4.70, 6.21] | |  |
| Machinery | 1.67 [1.13, 2.46] | 1.70 [1.15, 2.51] | | 1.57 [1.05, 2.33] | |  |
| All transportation | 1.48 [1.42, 1.54] | 1.56 [1.50, 1.62] | | 1.49 [1.43, 1.55] | |  |
| Nature/Environmental | 0.99 [0.92, 1.06] | 0.99 [0.92, 1.06] | | 0.99 [0.93, 1.07] | |  |
| Overexertion | 0.87 [0.83, 0.92] | 0.89 [0.85, 0.93] | | 0.87 [0.83, 0.91] | |  |
| Poisoning | 0.03 [0.00, 0.90] | 0.22 [0.00, 5.85] | | 0.02 [0.00, 0.85] | |  |
| Struck by/against | 0.91 [0.87, 0.95] | 0.92 [0.88, 0.96] | | 0.92 [0.87, 0.96] | |  |
| Other Specified | 1.08 [1.00, 1.17] | 1.10 [1.01, 1.19] | | 1.09 [1.00, 1.18] | |  |
| Unspecified | 0.97 [0.93, 1.01] | 0.98 [0.94, 1.02] | | 0.97 [0.93, 1.02] | |  |
| Multiple | 1.46 [1.37, 1.55] | 1.52 [1.43, 1.61] | | 1.46 [1.38, 1.56] | |  |
| **Body Region of Injury** |  |  | |  | |  |
| (ref. all other regions) |  |  |  |  |  |  |
| Head, face, and neck | 1.17 [1.13, 1.20] | 1.24 [1.20, 1.28] | | 1.16 [1.13, 1.20] | |  |
| Multiple regions | 1.29 [1.25, 1.33] | 1.40 [1.36, 1.44] | | 1.29 [1.25, 1.33] | |  |

Note:

Model 1: DTC vs. NTC discharge as a function of ICISS (continuous), and a two-way categorical-by-categorical interaction term of an indicator if the nearest hospital was DTC and time to the nearest DTC, adjusted by the main effects of all covariates and a quadratic effect of age

Model 2: DTC vs. NTC discharge as a function of three-way categorical-by-categorical interaction term of an indicator if the nearest hospital was DTC, time to the nearest DTC, and ICISS (categorical), adjusted by the main effects of all covariates and a quadratic effect of age

Model 3: DTC vs. NTC discharge as a function of ICISS (continuous), and a two-way categorical-by-continuous interaction term of an indicator if the nearest hospital was DTC and time to the nearest DTC, adjusted by the main effects of all covariates and a quadratic effect of age

**Appendix H**


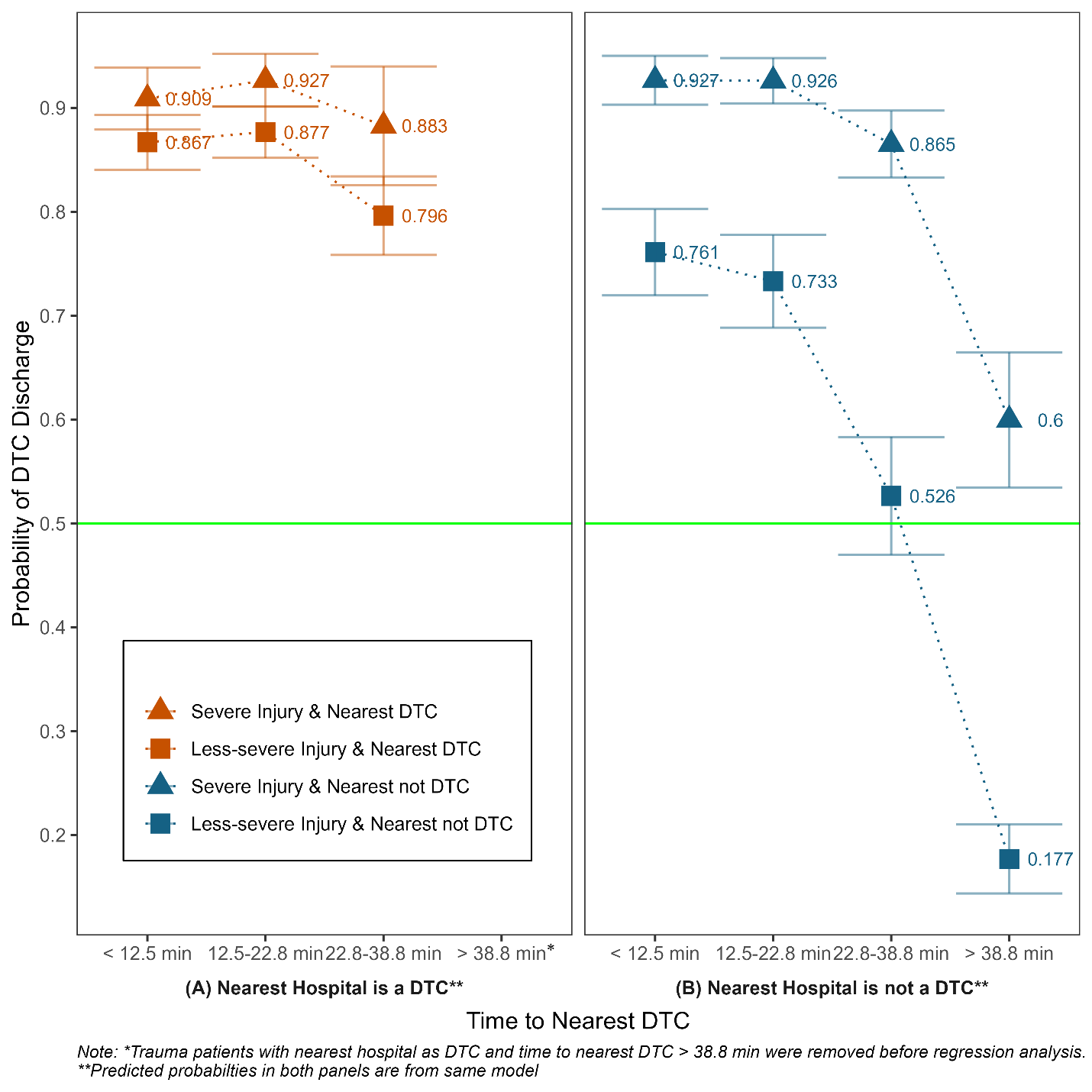


**Figure H.1: Predicted Probabilities of DTC Discharge by Injury Severity when using the ICISS cutoff of 0.941**

**Appendix I**


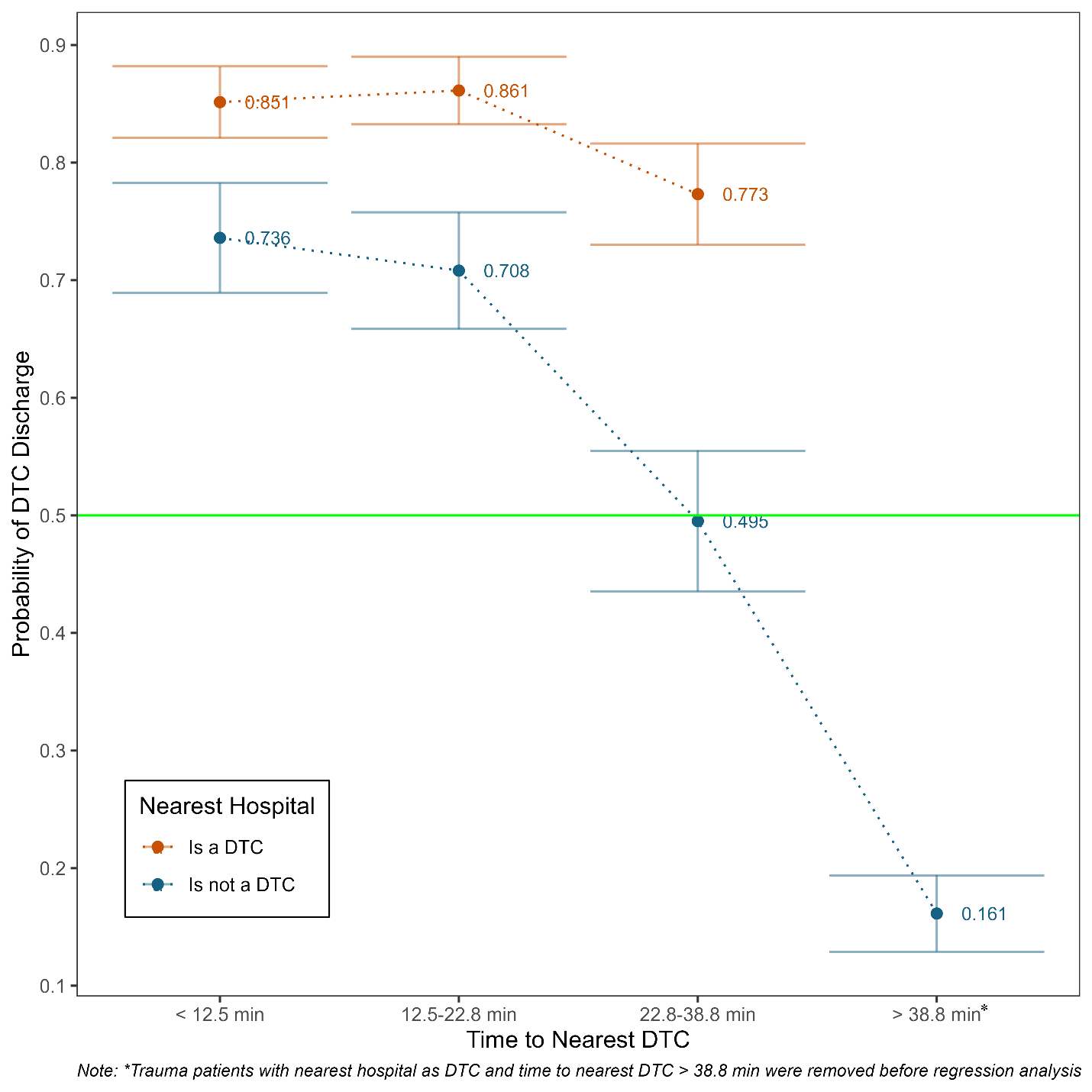


**Figure I.1: Predicted Probabilities of DTC Discharge, including samples with discharges from February to November 2021 only**


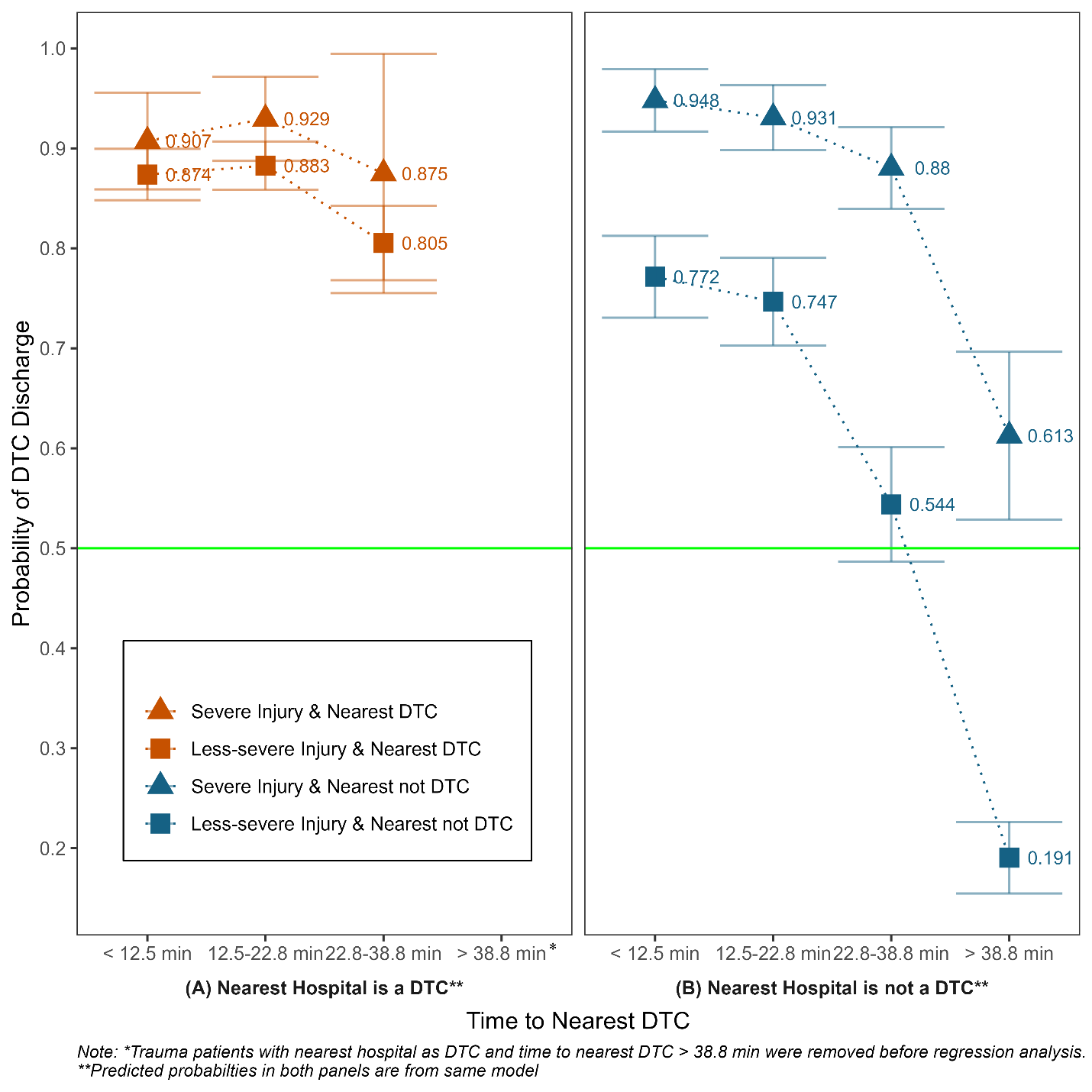


**Figure I.2: Predicted Probabilities of DTC Discharge by Injury Severity (ICISS cutoff 0.85), including samples with discharges from February to November 2021 only**

**Appendix J**


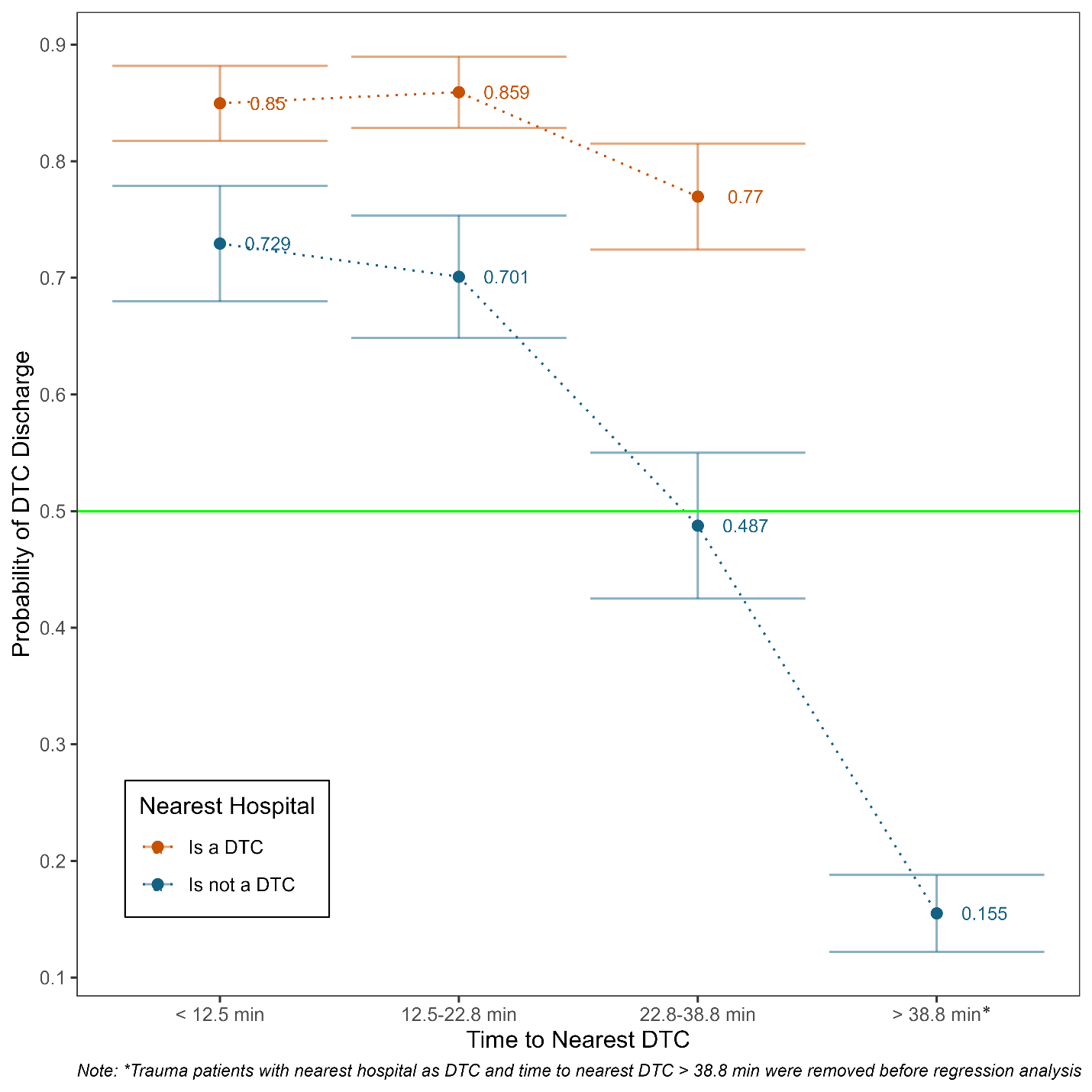


**Figure J.1: Predicted Probabilities of DTC Discharge, including samples discharged to residence only**


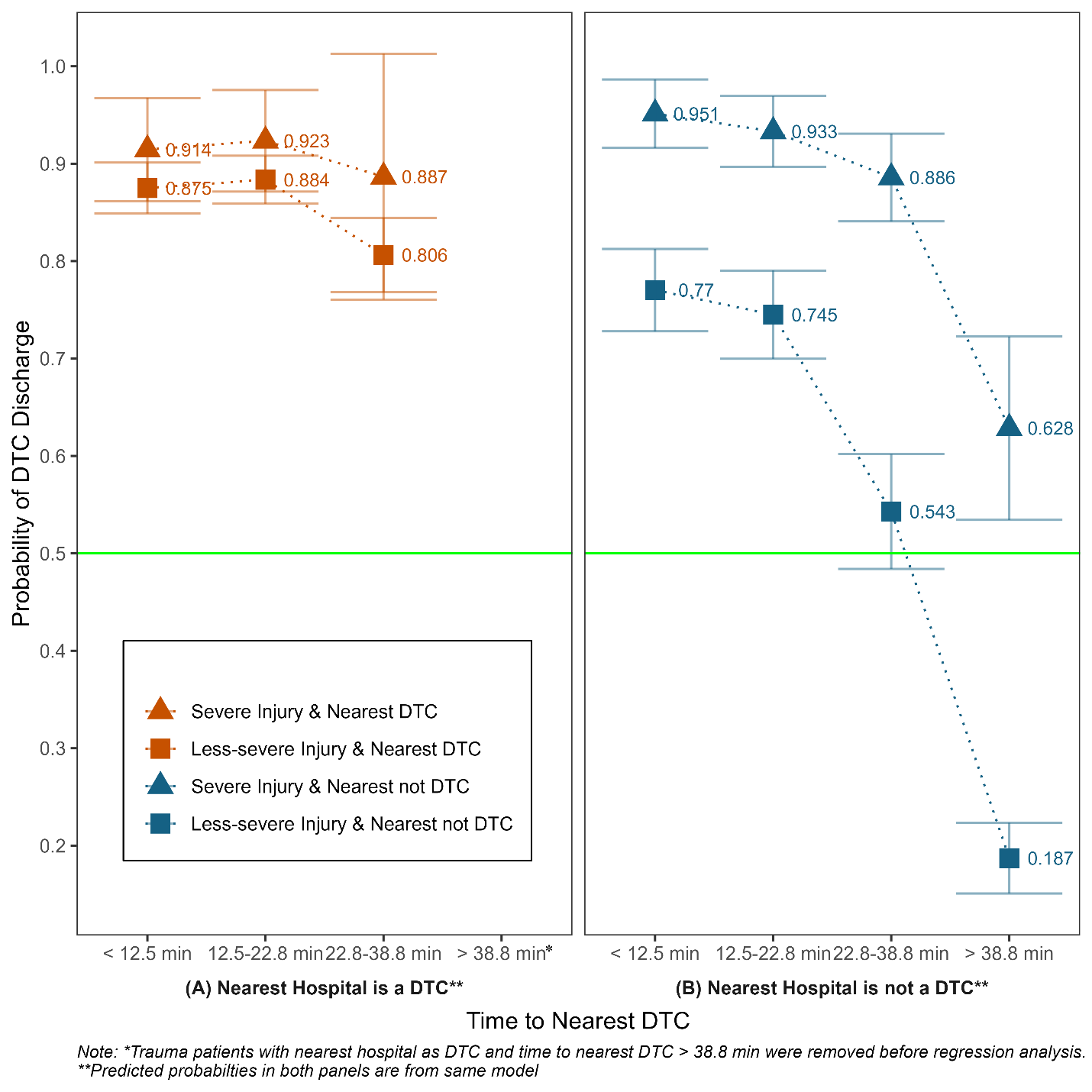


**Figure J.2: Predicted Probabilities of DTC Discharge by Injury Severity (ICISS cutoff 0.85), including samples discharged to residence only**

**Appendix K**


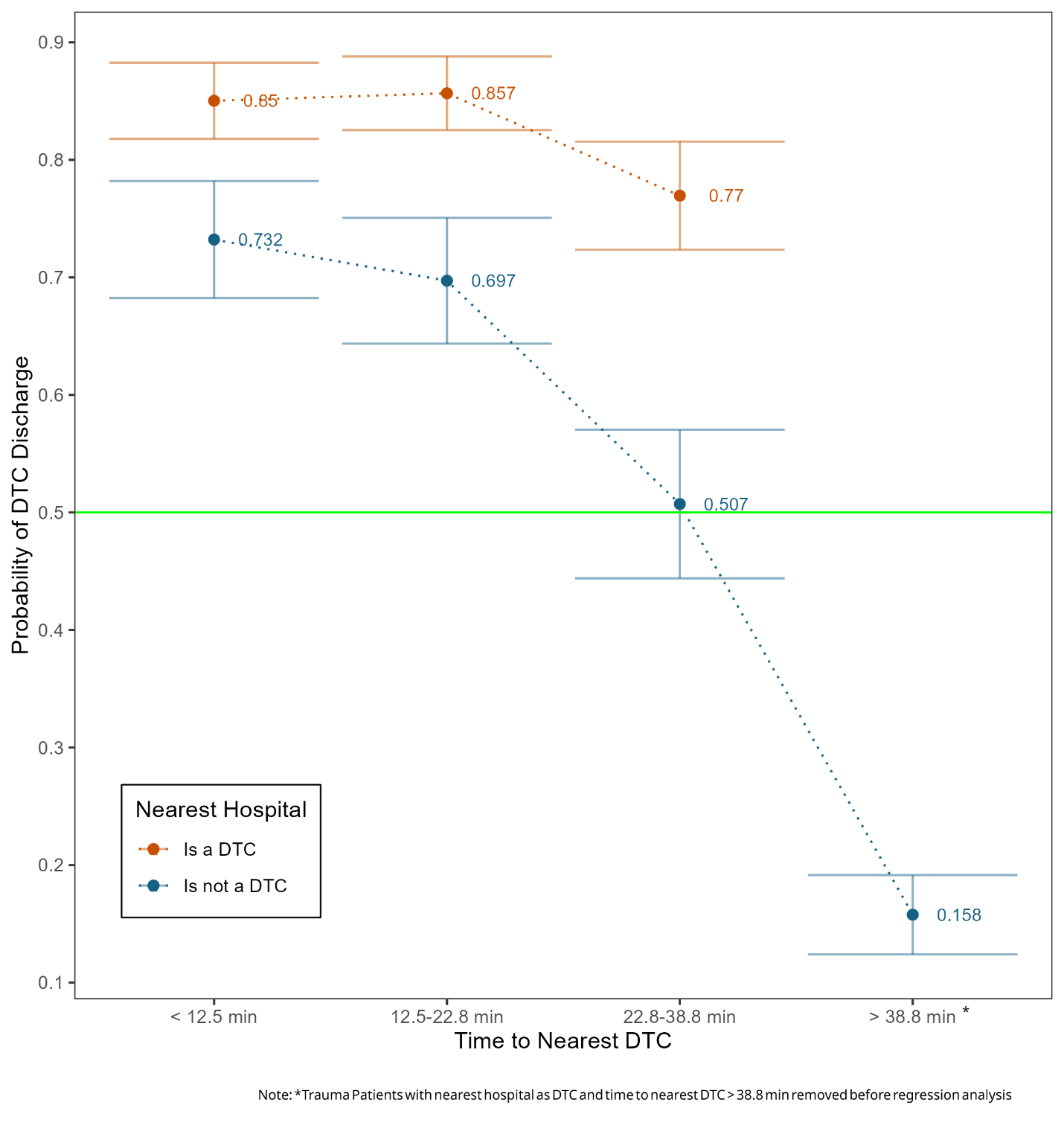


**Figure K.1: Predicted Probabilities of DTC Discharge excluding samples with “urgent” admit type**


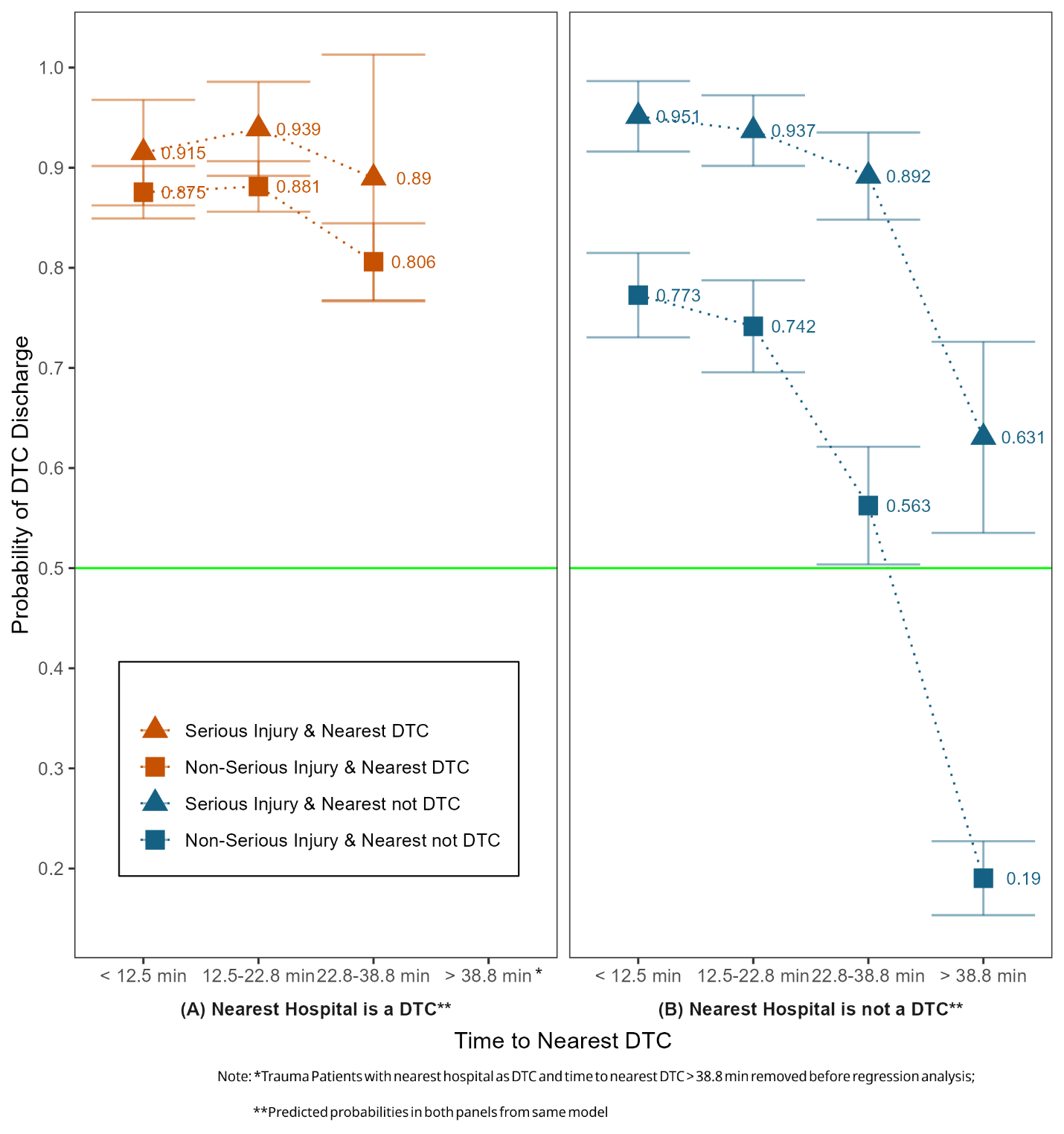


**Figure K.2: Predicted Probabilities of DTC Discharge by Injury Severity (ICISS cutoff 0.85), excluding samples with “urgent” admit type**
